# The Environmental Burden of the United States’ Bitcoin Mining Boom

**DOI:** 10.21203/rs.3.rs-5306015/v1

**Published:** 2024-10-22

**Authors:** Gianluca Guidi, Francesca Dominici, Nat Steinsultz, Gabriel Dance, Lucas Henneman, Henry Richardson, Edgar Castro, Falco J. Bargagli-Stoffi, Scott Delaney

**Affiliations:** 1. Department of Biostatistics, Harvard T.H. Chan School of Public Health, Boston, Massachusetts, USA; 2. Department of Computer Science, University of Pisa, Pisa, Italy; 3. WattTime.org, Oakland, California, USA; 4. *The New York Times*, New York, New York, USA; 5. Department of Civil, Environmental, and Infrastructure Engineering, Volgenau School of Engineering, George Mason University, Fairfax, Virginia, USA; 6. Department of Environmental Health, Harvard T.H. Chan School of Public Health, Boston, Massachusetts, USA

## Abstract

Bitcoin mines—massive computing clusters generating cryptocurrency tokens—consume vast amounts of electricity. The amount of fine particle (PM_2.5_) air pollution created because of their electricity consumption, and its effect on environmental health, is unknown. In this study, we located the 34 largest mines in the United States in 2022, identified the electricity-generating plants that responded to them, and pinpointed communities most harmed by Bitcoin mine-attributable air pollution.

From mid-2022 to mid-2023, the 34 mines consumed 32.3 terawatt-hours of electricity—33% more than Los Angeles—85% of which came from fossil fuels. We estimated that 1.9 million Americans were exposed to ≥0.1 μg/m^3^ of additional PM_2.5_ pollution from Bitcoin mines, which were often hundreds of miles away from communities they affected. Americans living in four regions—including New York City and near Houston—were exposed to the highest Bitcoin mine-attributable PM_2.5_ concentrations (≥0.5 μg/m^3^) with the greatest health risks.

## Introduction

Exposure to fine particle air pollution (PM_2.5_, particles with aerodynamic diameter ≤2.5 μm) from coal- and natural gas-fired electricity generating units (i.e., fossil fuel power plants) is associated with increased premature mortality and other adverse health outcomes^1^. As a result, policymakers have sought to curb reliance on fossil fuel power plants, thereby reducing public health harm from the air pollution they emit^2^. In the United States (U.S.), continuing progress could be hindered by the rapid rise of new, energy-intensive computing operations that require vast amounts of electricity, including companies that mine Bitcoin and other cryptocurrencies^3.4^.

In 2019, China dominated global Bitcoin mining, accounting for 65-75% of the total Bitcoin network^5^. In 2021, China banned cryptocurrency mines, partly given their prodigious electricity use, and many mining companies moved to the U.S.^6^ The share of global Bitcoin mining operations in the U.S. grew rapidly, from 4.5% in 2020 to 37.8% by January 2022, making it the world’s largest Bitcoin mining hub^7^. This explosion of growth and new electricity consumption led to an increase in electricity production and attendant emission of hazardous air pollutants by fossil fuel power plants, including PM_2.5_ pollution and its gaseous precursors.

Multiple studies have estimated greenhouse gas emissions from Bitcoin mining, with estimates ranging from tens of millions to over 100 million metric tons of CO_2_ per year^8,9^. However, to our knowledge, no peer-reviewed study has: (1) quantified the PM_2.5_ pollution attributable to Bitcoin mining in the U.S.; (2) identified specific Bitcoin mines and power plants that are responsible for the additional ambient PM_2.5_ pollution; and (3) pinpointed communities most affected by Bitcoin mine-attributable PM_2.5_ pollution. A comprehensive understanding of each of these issues will establish accountability and inform effective environmental and public health.

In this study, we estimated how much additional PM_2.5_ pollution was generated by the U.S. Bitcoin mining expansion that occurred from mid-2022 to mid-2023, and we pinpointed the communities that were exposed to it. Our analyses entailed the following steps.

First, we built a novel dataset of the 34 largest U.S. Bitcoin mines during the study period, their locations, and their power capacities. This dataset was compiled from in-depth investigative reporting, financial disclosures, land records, satellite imagery, and interviews. Further details are in Supplementary Information. Second, we identified the 635 power plants across the U.S. that supplied electricity in response to increased demand from each of the 34 mines during the study period. Third, we estimated the resulting air pollution emissions from each responding power plant that were attributable to Bitcoin mine electricity consumption. Fourth, we leveraged an atmospheric model to track the dispersion of each power plant’s Bitcoin mine-attributable air pollution emissions. Finally, we pinpointed communities most affected by Bitcoin mine-attributable PM_2.5_ pollution and quantified their annual average exposure to mine-attributable PM_2.5_ pollution.

## Results

### Bitcoin mine characteristics

The study period for our analysis was August 2022 to July 2023 Our sample included 34 Bitcoin mines in the U.S., with an aggregate capacity of 3,910 megawatts (MW). The U.S. Energy Information Administration (EIA) estimated that as of March 2023, the total power capacity of all 137 Bitcoin mines in the U.S. was 3,000-4,000 MW^10^. Therefore, our sample of 34 Bitcoin mines represented the largest power capacity mines in the U.S. (see Supplementary Information). The mines in our sample ranged in capacity from 38 MW (CleanSpark; College Park, GA) to 450 MW (Riot Digital; Rockdale, TX), and the median mine capacity was 100 MW (Table 1).

We estimated that these mines consumed 32.3 terawatt-hours (TWh) of electricity during the study period after accounting for periods when the mines were offline (see Supplementary Information for details). The electricity usage of these 34 mines would equal the annual demand of three to six million homes^11^.

**Figure 1.**
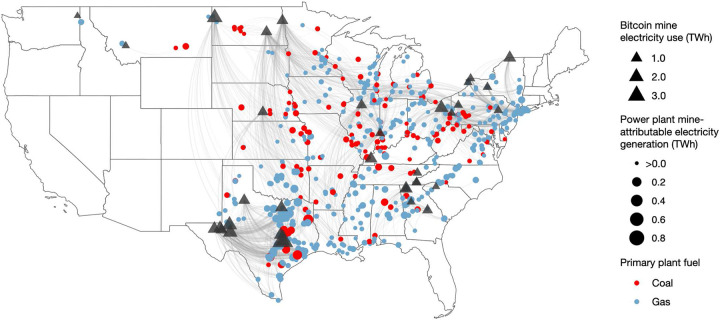
Bitcoin mines and the fossil fuel-fired power plants that generate electricity in response to them, August 2022-July 2023. ^1^ Electricity use in terawatt-hours (TWh) for each of the 34 mines in our sample is based on each mine’s confirmed capacity during the study period and the estimated percentage of time during the study period that the mine operated at full capacity, i.e., its uptime. These are lower-bound estimates. Some mines may have increased their capacity (and thus their electricity use) beyond their confirmed capacity during the study period. ^2^ Gray lines connect Bitcoin mines to the fossil fuel-fired power plants that generate additional electricity in response to each mine’s additional electricity consumption. They are based on the grid balancing authority in which each mine is located, and they also account for instances in which electricity is transferred between balancing authorities. Renewable generating plants (e.g., solar, hydro, wind) that respond to Bitcoin mine demand are not listed because they do not emit PM_2.5_ pollution. Responding power plants are often hundreds of miles from Bitcoin mines. ^3^ Each power plant’s mine-attributable electricity generation is the total electricity in TWh that each power plant generated during the study period in response to the electricity consumed by the Bitcoin mines.

The Bitcoin mining company Core Scientific operated more mines (seven mines with 814 MW aggregate capacity) than any other company. Mines were located in 15 U.S. states (Fig. 1). Ten mines were in Texas—more than in any other state—including three of the four mines in our sample with capacities of at least 200 MW. New York and Georgia hosted four mines each, while Pennsylvania and North Dakota each hosted three. No other state had more than one mine.

### Bitcoin mine-associated power plants

Each Bitcoin mine was located within a geographic area—a “*balancing authority region*”^12^—where electricity generation is constantly calibrated to match electricity consumption. There are 66 such regions in the contiguous U.S., varying in size from thousands to millions of square miles. When a Bitcoin mine begins operations or increases capacity, it demands more electricity, and power plants within the mine’s balancing authority region must respond to this increased “marginal” demand by increasing the electricity it generates. Within each balancing authority region, some power plants will generate proportionately more electricity than others in response to increased demand. The relative contribution of each power plant in response to increased marginal demand is determined by several factors, including power plant fuel type and capacity, and whether renewable electricity sources are available.

We estimated how much electricity the 635 individual power plants generated in response to consumption by each of the 34 Bitcoin mines during the study period using data from the EIA and the U.S. Environmental Protection Agency (EPA) and regression models from WattTime. See Supplementary Information for details. Fossil fuel power plants generated 85% of the increased electricity demand from Bitcoin mines, including 138 coal-fired and 497 natural gas-fired plants. Bitcoin mines and the power plants that increased generation in response to them were often separated by multiple states and hundreds of miles (Fig. 1). For example, electricity consumed by the Atlas Power Bitcoin mine in Williston, ND, induced a large response from the Jeffrey Energy Center coal-fired power plant located 722 linear miles and three states away in St. Marys, KS (Table 1).

### Bitcoin mine-attributable power plant emissions

After establishing the amount of electricity each power plant generated in response to each Bitcoin mine’s electricity use, we quantified the PM_2.5_ and CO_2_ emissions from each plant that were attributable to Bitcoin mining operations using data from the U.S. EPA (see Supplementary Information). Table 1 reports Bitcoin mine-attributable power plant PM_2.5_ emissions for the 10 mines in our sample responsible for the largest amount of primary PM_2.5_ emissions. See Supplementary Information for results from all 34 mines.

Mine electricity consumption, PM_2.5_ emissions, and CO_2_ emissions were only loosely correlated because the mix of power plants that responded to each mine’s electricity demand was often different, and some plants emitted more PM_2.5_ and CO_2_ per TWh than others. For example, the Core Scientific mine in Calvert City, KY, was responsible for more PM_2.5_ emissions than any other mine despite ranking 8th in electricity consumption and 11th in attributable CO_2_ emissions (Table 1). This discrepancy is partly because the Calvert City Core Scientific mine induced a large response from the Shawnee Fossil Plant in West Paducah, Kentucky, which is a coal-fired plant with fewer pollution control than similar plants. The Shawnee Fossil Plant also responded to electricity consumed by the BitDeer mine in Knoxville, TN, which, despite ranking 24th in electricity consumption, was responsible for the 4th highest PM_2.5_ emissions (Table 1).

### Environmental impact of Bitcoin mine-attributable PM_2.5_ air pollution

Power plants emit several air pollutants,including PM_2.5_ emitted directly from their stacks (i.e., “*primary*” PM_2.5_), NO_x_, and SO_x_. Once airborne, NO_x_ and SO_x_ can react in the atmosphere to form additional (i.e., “*secondary*”) PM_2.5_ pollution^13^. To assess ambient (i.e., primary and secondary) PM_2.5_ air pollution across the U.S. attributable to Bitcoin mines, we used the InMAP model, which is a computationally efficient chemical transport model developed to trace air pollution from specific sources^14^. We aggregated all InMAP model estimates of total Bitcoin mine-attributable ambient PM_2.5_ air pollution to the Census tract level, mapped the resulting concentrations, and quantified the number of people exposed to it.

**Figure 2.**
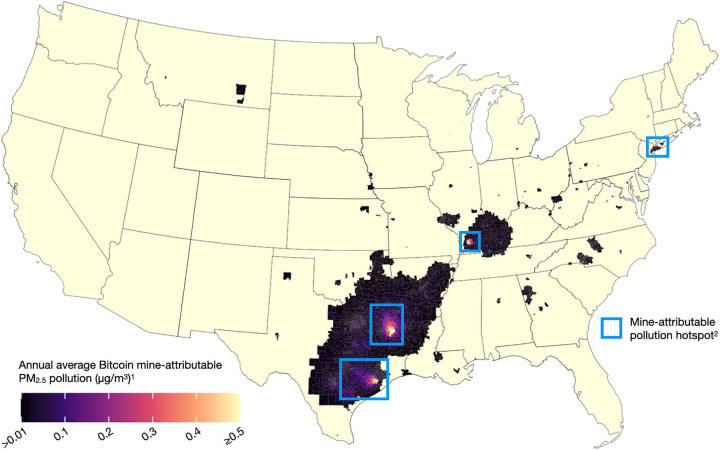
Additional ambient PM_2.5_ pollution attributable to Bitcoin mines, August 2022-July 2023. ^1^ Mapped are annual average ambient PM_2.5_ concentrations attributable to the 34 Bitcoin mines in our sample. Thus, these are estimates of *marginal* (i.e., additional) pollution attributable to Bitcoin mine electricity use, not estimates of total air pollution from all sources. Estimates are from the InMAP model and account for (1) primary PM_2.5_, NO_x_, and SO_x_ emissions from specific power plants responding to increased demand for electricity induced by Bitcoin mines; (2) power plant attributes (e.g., location, stack height); and (3) meteorology. Polluted census tracts (i.e., tracts displayed in any color other than the pale-yellow base color) are those with average annual mine-attributable PM_2.5_ concentrations of at least 0.01 μg/m^3^. Tracts in bright yellow may exceed 0.5 μg/m^3^. See Table 2. ^2^ Blue squares approximate the 4 mine-attributable PM_2.5_ “hotspots.” Hotspots are empirically defined as contiguous counties containing at least one census tracts with an ambient Bitcoin mine-attributable PM_2.5_ concentration ≥ 0.10 μg/m^3^. See Figure 3.

Extensive prior research demonstrates that even small increases in long-term PM_2.5_ air pollution increase the risk of premature mortality and other adverse health outcomes^16,17^. We estimated that 46,211,621 Americans living in 27 states were exposed to measurable (i.e., ≥0.01 μg/m^3^ on average) concentrations of Bitcoin mine-attributable PM_2.5_ pollution from August 2022 through July 2023 (Fig. 2). This exposure was in addition to PM_2.5_ pollution attributable to all other sources. Americans exposed to Bitcoin mine-attributable pollution lived in regions extending west to Montana, east to New York, and south to Texas. Both rural and urban regions were affected, including densely populated areas in or near New York City; Houston, Austin, and San Antonio, Texas; New Orleans and Shreveport, Louisiana; Little Rock, Arkansas; St. Louis, Missouri; Evansville, Indiana; Nashville, Tennessee; Atlanta, Georgia; and Greensboro and Charlotte, North Carolina.

**Figure 3.**
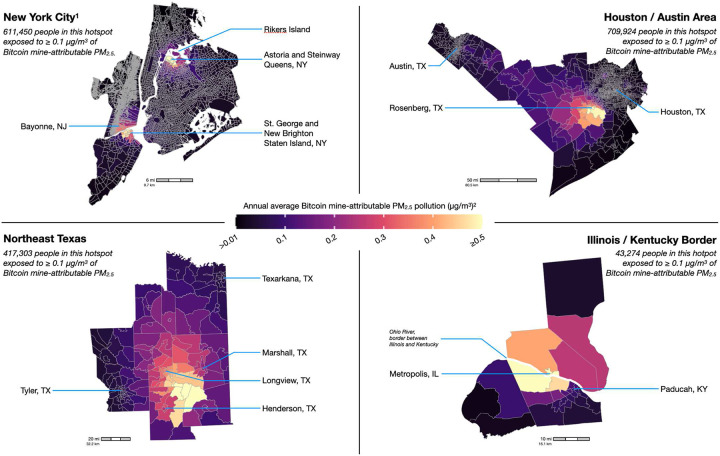
Bitcoin mine-attributable PM_2.5_ pollution hotspots in detail, August 2022-July 2023. ^1^ Mapped are U.S. census tracts (2020 Census borders) within hotspots. Hotspots are empirically defined as contiguous counties containing at least one census tract with a total (i.e., primary and secondary) Bitcoin mine-attributable PM_2.5_ concentration ≥0.10 μg/m^3^. A fifth hotpot matching this definition near San Antonio, TX, is excluded because it is considerably smaller and its Bitcoin mine-attributable PM_2.5_ concentrations are considerably lower than those in the four hotspots detailed here. See Table 2 for specific concentrations in selected hotspot census tracts along with a list of power plants and Bitcoin mines primarily responsible for the Bitcoin mine-attributable PM_2.5_ pollution. ^2^ Estimates are from InMAP. They represent the additional ambient PM_2.5_ pollution that is attributable to Bitcoin mines. See Figure 2. While the power plants emitting this pollution are often near or within the hotspots, the Bitcoin mines ultimately responsible for the emissions are typically hundreds of miles away.

We further estimated that 1,904,959 Americans across five states were exposed to Bitcoin mine-attributable PM_2.5_ concentrations of at least 0.10 μg/m^3^ on average across the study period. Most of these residents lived in 1 of 4 “*hotspots*,” which we defined as contiguous counties containing at least one Census tract with a total mine-attributable PM_2.5_ concentration ≥0.10 μg/m^3^ (Fig. 2). These are (1) New York City, (2) the Houston/Austin metropolitan area, (3) Northeast Texas, and (4) areas along the Illinois/Kentucky border (Fig. 3). Moreover, within each hotspot, Bitcoin mine-attributable PM_2.5_ concentrations in at least some tracts exceeded 0.50 μg/m^3^.

While many residents in these hotspots lived near fossil fuel power plants, the Bitcoin mines ultimately responsible for the additional mine-attributable PM_2.5_ pollution they inhaled were often hundreds of miles away. New York City, for example, had two primary areas exposed to high levels of mine-attributable air pollution. Residents in the first area, at the northern end of Staten Island, were exposed to Bitcoin mine-attributable PM_2.5_ concentrations as high as 0.67 ug/m^3^, which accounted for 8.7% of all PM_2.5_ air pollution over the area (Table 2). Most of this pollution came from the Bayonne Energy Center gas-fired plant across the Kill van Kull in New Jersey. However, the Bitcoin mines ultimately responsible for these emissions were in upstate New York. For example, the Coinmint mine in Massena, New York, located near New York’s border with Canada and the 7th largest mine by capacity in our sample, was 100 linear miles away from Bayonne, New Jersey, and the pollution it was responsible for in Staten Island, New York.

The second area of exceedingly high mine-attributable PM_2.5_ pollution in New York City was in Queens, near the natural gas-fired Astoria Energy power plants. Here, the annual average of Bitcoin mine-attributable PM_2.5_ concentration reached 0.59 μg/m^3^. This area also includes the Rikers Island jail complex (0.46 ug/m^3^), which housed approximately 6,000 prisoners and employed thousands more staff during the study period. Rikers Island has long been plagued by flooding, extreme heat, and poor air quality. Bitcoin-attributable PM_2.5_ adds yet another environmental exposure associated with adverse health outcomes.

We found two separate hotspots in Texas. The first stretched between Austin and Houston, with the highest Bitcoin mine-attributable PM_2.5_ concentrations near Houston’s southeast suburbs, including Sugar Land and Rosenberg (0.52 μg/m^3^). We estimated that 709,924 people were exposed to at least 0.10 ug/m^3^ of additional mine-attributable PM_2.5_ pollution in the Houston/Austin region, making it the largest hotspot by population. A large proportion of pollution in this hotspot came from the coal- and gas-fired W.A. Parish Generating Station (Richmond, Texas) and the Sam Seymour Power Plant (La Grange, Texas). While the Sam Seymour plant had SO_2_ scrubbers installed on two of its three stacks to reduce emissions, the W.A. Parish facility has no scrubbers on any of its four stacks, making its emissions particularly toxic. Recent research^15^ estimated that emissions from the W.A. Parish plant led to 3,500 (95% CI: 3,200–3,900) premature deaths among older adults in the U.S. from 1999 to 2020, largely before Bitcoin mining in Texas began. Two years later, our analysis found that Bitcoin mining was responsible for additional PM_2.5_ — up to 0.5 μg/m^3^ — from W.A. Parish that was inhaled by residents in the Austin/Houston region. The Bitcoin mines ultimately responsible for this increased PM_2.5_ air pollution were located hundreds of miles west of Austin, including the Riot Digital mine in Rockdale, Texas, the Cipher Mining mine in Odessa, Texas, and the U.S. Bitcoin mine in McCamey, Texas.

These West Texas mines also increased electricity demand from plants in other parts of Texas. Thus, they were also responsible for increased mine-attributable PM_2.5_ pollution in the Northeast Texas hotspot, where additional annual average PM_2.5_ concentrations reached as high as 0.86 μg/m^3^ near Tatum, Texas, and made up 11.4% of all PM_2.5_ air pollution in the area (Table 2). Areas around Tyler, Longview, and Texarkana, Texas, were also affected. A substantial proportion of this mine-attributable PM_2.5_ pollution was emitted by the Martin Lake power plant (Tatum, Texas) and the Sandy Creek Energy Station (Riesel, Texas), both fueled by coal. However, compared to Sandy Creek, the Martin Lake facility is larger and has no SO_2_ scrubbers installed. From 1999 to 2020, emissions from Martin Lake were associated with 4,100 (95% CI: 3,700–4,500) premature deaths among older Americans^15^. In our analysis, Bitcoin mines again increased the electricity demand—and thus hazardous air pollution emissions—produced by these fossil fuel power plants.

Another coal-fired plant—the Shawnee Fossil Plant in West Paducah, Kentucky—was responsible for a large proportion of Bitcoin mine-attributable PM_2.5_ pollution in the fourth hotspot along the Illinois/Kentucky border. This hotspot included the highest concentration of mine-attributable PM_2.5_ pollution anywhere in the U.S.: 0.96 μg/m^3^ in Metropolis, Illinois. We estimated that 13.1% of all PM_2.5_ air pollution over parts of Metropolis, Illinois, during the study period was attributable to Bitcoin mines (Table 2). The mines responsible for these emissions were spread across at least three states and included the BitDeer mine in Knoxville, Tennessee, the Core Scientific mine in Calvert City, Kentucky, and the Core Scientific mine in Marble, North Carolina.

The Core Scientific mine in Marble, North Carolina, illustrates the importance of mine location and electricity utility provider in determining the location and amount of Bitcoin mine-attributable PM_2.5_ pollution. Electricity for the Core Scientific mine was supplied by two separate utilities that were part of different balancing authorities. Specifically, 35 MW of the Marble mine’s 104-MW total capacity was supplied by TVA Murphy Power, which was balanced by the Tennessee Valley Authority (TVA). Marginal increases in electricity consumption within the TVA induced additional generation at the Shawnee Fossil Plant and thus increased mine-attributable PM_2.5_ pollution over the Illinois/Kentucky border. However, the Marble mine’s other 69 MW of capacity were balanced by Duke Energy Carolinas and thus induced electricity generation (and attendant pollution) at a different mix of power plants elsewhere.

Within each hotspot, we also explored possible Bitcoin mine-attributable PM_2.5_ exposure inequities. In many regions, socially marginalized groups experience higher air pollution exposure than others. In this study, we investigated whether Census tracts with higher proportions of non-White or low-income residents experienced higher levels of PM_2.5_ exposure from Bitcoin mining than tracts with more White or higher-income residents. We found no systemic exposure inequities in any of the four hotspots (see Supplementary Information).

## Policy Implications

Bitcoin mines, which are unregulated in the U.S., are an emerging and significant challenge to U.S. environmental health and air pollution regulation for two reasons. First, mining Bitcoin requires enormous amounts of electricity^9^. Bitcoin mines induce electricity production mainly from fossil fuel power plants, including from some of the country's dirtiest coal-fired plants. This increased demand hinders policy efforts to retire fossil fuel power plants and reduce air pollutant emissions. Furthermore, additional Bitcoin mine-attributable PM_2.5_ emissions could slow efforts to attain the new National Ambient Air Quality Standard for PM_2.5_ of 9 μg/m^3^ recently announced in early 2024^2^.

Second, air pollution regulatory efforts are further complicated because Bitcoin mines in one state often induce air pollution in other states, leaving residents in affected states with no state-based political power to reduce the Bitcoin mine-attributable air pollution they breathe. For example, residents in Metropolis, Illinois, breathe high concentrations of Bitcoin mine-attributable PM_2.5_ air pollution, yet the Illinois state government has no power to regulate the siting of Bitcoin mines in North Carolina, which induce electricity generation in Kentucky and result in air pollution in southern Illinois. As a result, federal regulation is required to address the impact of air pollution from the Bitcoin mining boom.

## Future Research

While this paper provides a foundation for understanding Bitcoin mining’s environmental impact, our estimates of the present impact of Bitcoin mining operations in the U.S. are very conservative for several reasons. First, our sample underrepresents the total number of U.S. Bitcoin mines. Second, the mines we examined have likely increased their power usage since the end of our study period in July 2023. For example, the BitDeer mine in Rockdale, Texas, is already more than 2.5 times larger than it was during the study period, and the global Bitcoin network hash rate (a measure of the computational power used to mine and process transactions on a cryptocurrency network) increased by 105% over the course of 2023^18^. Furthermore, the state of Texas has seen a dramatic rise in its share of the U.S. Bitcoin mining hash rate, growing from 8.43% at the end of 2021 to 28.50% as of July 2023. This indicates a growing demand for Bitcoin mining and is attributed to Texas’ pro-crypto policy environment^19^.

If all mines met their proposed capacity increase (see Supplementary Information), the total capacity would increase from 3,910 MW to 7,945 MW, representing a 103% increase. To account for this rise in the energy demand, future research should expand the dataset to include more mines and update power capacities in this rapidly evolving industry.

Finally, the increase in toxic PM_2.5_ air pollution induced by Bitcoin mining will negatively impact human health. PM_2.5_ increases mortality, morbidity, and hospitalization risk. Air pollution from coal-fired power plants (coal PM_2.5_) is particularly toxic, with a mortality risk 2.1 times greater than that of PM_2.5_ from other sources. Thus, a holistic assessment of the impact of Bitcoin mines should consider their health impact along with the environmental impact.

## Methods

### Bitcoin data

Data on the Bitcoin mining facilities was collected from *The New York* Times^20^. *The New York Times* identified the 34 largest Bitcoin miners in the U.S. as of March 2023, defined as mines with capacities near or exceeding 40 megawatts. Data collected included Bitcoin miners’ proposed power capacity, their actual power capacity, their uptime—i.e., the percentage of the total power capacity at which the miners are assumed to have worked in the year under study—the exact locations (latitude and longitude), and balancing authorities from where they source electricity. *The New York Times* investigation pinpointed major Bitcoin mines by scrutinizing public statements, news articles, financial disclosures, and by conducting interviews with mine operators. Subsequently, satellite imagery and land records were employed to ascertain the precise location of each operation and its connection to a specific part of the U.S. electric grid. After completing its investigation, *The New York Times* presented its findings to each Bitcoin mining operation in order to allow the operations to comment and make corrections.

### Power plants data

Data on power plants was retrieved from WattTime, the U.S. EPA, and the EIA. WattTime—an environmental nonprofit tech that specializes in measuring and monitoring greenhouse gas emissions—harnessed and leveraged data from a variety of sources, including the U.S. EPA, EIA, grid operators, and a machine learning algorithm, to provide, for each balancing authority grid region, power plants’ exact locations, their marginal contribution to supply a marginal MWh of demand (i.e., the “marginal operating emissions rate,” or MOER), at daily level, in their balancing authority grid region of competence, and their amount of CO_2_, NO_x_, SO_2_, and PM_2.5_ emissions per MWh produced (see Supplementary Information for more details)^21^.

We relied on the U.S. EPA’s 2022 eGRID data to retrieve power plant energy fuels^22^.

### Characteristics of affected communities

We used demographic data from the American Community Survey 5-year estimates (ACS-5) at the Census tract level^23^. The ACS covers a broad range of variables related to the U.S. population’s social, economic, demographic, and housing characteristics. We used ACS-5 data from 2022 for our socio-demographic analyses^24^.

### Quantifying Bitcoin-attributable emissions

This study estimates the additional exposure to PM_2.5_ due to the additional energy demand from Bitcoin data mining facilities. We tracked each power plants’ contributions to ambient PM_2.5_ and averaged concentrations at the census tract level to study the exposed populations’ demographics. We retrieved from *The New York Times* the location and power capacity of each Bitcoin miner and quantified Bitcoin miner-attributable emissions as follows.

We first estimate the energy demand for year y by multiplying the power capacity (MW) of each Bitcoin miner i, by their uptime factor, and finally by the number of hours in a year (8760).


MinerEnergyDemand(i,y)=PowerCapacity(i,y)⋅Uptime(i,y)⋅8760.

We assume that the energy demand for each miner is constant throughout the year.

Second, we link each Bitcoin miner to its respective Balancing Authority. This is done by cross-checking Bitcoin mining facilities’ locations, Balancing Authorities' borders (polygons), and media and news documents assigning specific Bitcoin miners’ energy demands to specific utilities or balancing authorities.

To find each power plant’s load increase due to the Bitcoin miners’ energy demand, we divide the yearly energy demand of each miner among the supplying power plants within the grid region of the miner, according to their respective MOERs, described earlier. Given miner i, year y, power plant j, and the balancing authority B, we have

PowerPlantLoad(i,y,j,B)=MinerEnergyDemand(i,y)⋅MOER(y,j,B)

Where: j∈B, that is, power plant j belongs to the balancing authority

B; MOER(i,y,B) represents the percentage contribution of power plant i to the production of energy in a specific balancing authority B in year y, such that the sum of all power plants’ contributions within a balancing authority B equals one,

$$\sum_{j=1}^{N_{b}}\;MOER(j,B)=1$$

where $N_{B}$ is the number of power plants in the balancing authority B.

These computations yield the proportion of megawatt-hours (MWh) supplied by each power plant to each Bitcoin facility.

Third, we multiply the resulting marginal MWh load borne by each power plant due to the miner’s operations by the power plant’s emissions per MWh coefficient. This coefficient varies for each power plant depending on its fuel type and efficiency (e.g., power plants that rely on coal typically have higher primary PM_2.5_ emissions per MWh than gas plants).


PowerPlantEmissions(i,y,j,B)=PowerPlantLoad(i,y,j,B)⋅EmissionperMWh(y,j,B).

Emissions coefficients are developed separately for each emitted pollutant (CO_2_, NO_x_, SO_2_, and PM_2.5_) and are unique to each power plant.

In summary, this entire data pipeline identifies the annual emissions that each plant produces due to the Bitcoin miner’s marginal electricity demand. Sensitivity analyses to our estimates—to assess the robustness of our model—have been produced. Results are reported in the Supplementary Information.

### Tracking air pollution dispersion and atmospheric transformation

To quantify the dispersion of PM_2.5_ emissions produced by each power plant due to Bitcoin mining operations, we employed the Intervention Model for Air Pollution (InMAP), a reduced-complexity model developed for the U.S. that estimates annual average ambient PM_2.5_ concentrations^14^. More specifically, the InMAP model estimates annual exposure to ambient PM_2.5_, using a variable spatial resolution depending on the population density, ranging from squares with sides as short as 1 km in urban areas to larger ones in rural areas.

InMAP is a reduced complexity model that approximates scientists’ full understanding of atmospheric transport, dispersion, and chemistry using a simpler algorithm than full-scale chemical transport models. We chose this model because the computational demand of full-scale models would make infeasible calculations of specific power plants’ contributions to ambient PM_2.5_. Evaluations have shown InMAP has good performance in predicting changes in PM_2.5_, sulfate, and secondary organic aerosol concentrations compared with other air quality models^25,26^.

InMAP takes as inputs the location (latitude and longitude) of each point emission source (i.e., the power plants) and the quantities of emissions (PM_2.5_, SO_x_, and NO_x_). We used our SO_2_ estimates as a proxy for SOx—SO_2_ is a sufficient indicator for the larger group of gaseous sulfur oxides SO_x_, especially in coal power plants^25^. The output grid with average PM_2.5_ concentrations is then overlaid onto the U.S. Census tracts. Grid cells’ PM_2.5_ exposure values are transposed to Census tract exposure values by averaging the values of the grid cells overlapping each Census tract. Grid cells overlapping multiple tracts have their PM_2.5_ concentrations equally divided between the tracts.

With Census tract exposures, we identified and classified all the contiguous counties as hotspots containing at least one census tract with an ambient Bitcoin mine-attributable PM_2.5_ concentration ≥ 0.10 μg/m^3^.

### Demographic impact

To investigate potential income and racial and ethnic exposure disparities within each hotspot, we created separate scatter plots of tract-level Bitcoin-attributable PM_2.5_ concentrations against (1) the percentage of non-White residents and (2) the median household income for every Census tract of each hotspot.

To make results comparable across different hotspots, we converted the demographic values (i.e., for each tract, the percentage of non-White residents and the median household income) to percentile ranks within each hotspot, with ranked bounded as >0% and <100%. For example, the tracts in the Northeast Texas hotspot with the highest median incomes were designated as the 99th percentile or higher, while the tract with a median income at the median for the Northeast Texas hotspot was designated as the 50th percentile. Resulting scatter plots illustrate whether lower income tracts (or tracts with proportionately more non-White residents) experienced higher levels of Bitcoin mine-attributable air pollution.

## Figures and Tables

**Table 1.** Ten largest Bitcoin mines by estimated annual mine-attributable PM_2.5_ emissions, August 2022-July 2023.

		Bitcoin mine-attributable emissions^1^				Annual mine electricity use^2^		Power plant producing the most PM_2.5_ pollution attributable to each mine^3^			
		PM_2.5_		CO_2_							
Mine Operator	Mine Location	Tons	Rank	kTons	Rank	TWh	Rank	Plant Name	Approx. Location	Fuel	Miles from mine
Core Scientific	Calvert City, KY	134	1	548	11	1.25	8	Shawnee Fossil Plant	West Paducah, KY	Coal	22
Riot Digital	Rockdale, TX	99	2	2020	1	3.74	1	Martin Lake Power Plant	Tatum, TX	Coal	188
Viking	Akron, OH	61	3	708	6	1.25	8	Big Sandy Power Plant	Louisa, KY	Gas	208
BitDeer	Knoxville, TN	54	4	219	24	0.50	24	Shawnee Fossil Plant	West Paducah, KY	Coal	286
Core Scientific	Marble, NC	51	5	429	17	0.87	14	Shawnee Fossil Plant	West Paducah, KY	Coal	303
Coinmint	Massena, NY	50	6	427	18	1.29	7	Bayonne Energy Center	Bayonne, NJ	Gas	100
Atlas Power	Williston, ND	46	7	1142	2	2.00	2	Jeffrey Energy Center	St. Marys, KS	Coal	722
Cipher Mining	Odessa, TX	44	8	900	3	1.72	3	Martin Lake Power Plant	Tatum, TX	Coal	454
US Bitcoin	McCamey, TX	39	9	793	4	1.66	4	Martin Lake Power Plant	Tatum, TX	Coal	456
BitDeer	Rockdale, TX	38	10	763	5	1.41	6	Martin Lake Power Plant	Tatum, TX	Coal	188

1 Bitcoin mine-attributable emissions are per year. These are estimates based on (1) annual mine electricity use; (2) the grid balancing authority subregion(s) to which each mine is connected; and (3) marginal stack emissions from power plants that respond to increased electricity load within each balancing authority subregion. Ranks are within our sample of 34 mines. CO_2_ emissions are in kilotons (kTons). PM_2.5_ emissions are in tons, do not account for secondary PM_2.5_ (i.e., they are primary PM_2.5_ emissions only), and do not account for atmospheric dispersion. Ambient Bitcoin mine-attributable PM_2.5_ concentrations (i.e., combined primary and secondary PM_2.5_) in μg/m^3^ across the U.S. are distinct and detailed separately in Fig. 2, Fig. 3, and Table 2.

2 Annual mine electricity use is in terawatt-hours (TWh). It is based on (1) Bitcoin mine capacity in megawatts (MW) and (2) estimated annual uptime (i.e., the percent of time the mine is operating at capacity). Ranks are within our sample of 34 mines.

3 Power plants emitting the most Bitcoin mine-attributable PM_2.5_ pollution related to each mine are identified based on the grid balancing authority subregion in which each mine is located. Within each subregion, many power plants supply electricity in response to a marginal increase in electricity demand (i.e., load). Plants listed here are those responsible for the largest amount of Bitcoin mine-attributable PM_2.5_ emissions in tons. Miles from mine are calculated as the approximate linear distance between each Bitcoin mine and corresponding power plant.

**Table 2.** Selected communities with the highest estimated Bitcoin mine-attributable annual PM_2.5_ exposure and the Bitcoin mines and power plants primarily responsible for it, August 2022-July 2023, by hotspot^1^

Responsible Bitcoin mines^2^		→	Responsible power plants^3^			→	Selected affected communities^4^				
*Mines primarily responsible for each hotspot's* *Bitcoin mine-attributable PM_2.5_ pollution* *(name, location)*			*Plants primarily responsible for each hotspot's Bitcoin mine-* *attributable PM_2.5_ pollution (name, location, fuel)*				*City / Region*	*County*	*Census* *Tract ID*	*Tract* *Population*	*Increased PM_2.5_* *from Bitcoin* *mines (μg/m^3^)*
**Hotspot: New York City**											
Coinmint	Massena, NY		Bayonne Energy Center	Bayonne, NJ	Gas		**Staten Island, NY**	Richmond	7	4583	**0.67**
Greenidge Generation	Dresden, NY								77	1969	**0.47**
Terawulf	Somerset, NY								81	4638	**0.41**
Digihost	North Tonawanda, NY	→	Astoria Energy^5^	Queens, NY	Gas	→	**Queens, NY**	Queens	123.01	3107	**0.59**
					Gas				111	2949	**0.52**
									113	4466	**0.52**
							**Rikers Island, NY**	Bronx	1	~6000^6^	**0.46**
**Hotspot: Houston Area**											
Riot Digital	Rockdale, TX		W.A. Parish Generating Station	Richmond, TX	Coal/Gas		**Rosenberg, TX**	Fort Bend	6755.02	17681	**0.52**
Cipher Mining	Odessa, TX	→	Sam Seymour Power Plant	La Grange, TX	Coal	→	**Sugar Land, TX**		6755.01	7115	**0.52**
US Bitcoin	McCamey, TX								6755.03	7269	**0.49**
**Hotspot: Northeast Texas**											
Riot Digital	Rockdale, TX		Martin Lake Power Plant	Tatum, TX	Coal		**Longview, TX**	Gregg	105.01	2420	**0.53**
Cipher Mining	Odessa, TX		Sandy Creek Energy Station	Riesel, TX	Coal				105.02	2834	**0.46**
US Bitcoin	McCamey, TX								15	4252	**0.45**
Bitdeer	Rockdale, TX	→				→		Harrison	206.03	7299	**0.45**
Rhodium Enterprises	Temple, TX								206.04	4440	**0.44**
Core Scientific	Pescos, TX								206.06	5974	**0.41**
Genesis Digital Assets	Pyote, TX						**Tatum, TX**	Rusk	9501.01	3159	**0.86**
Core Scientific	Denton, TX								9501.02	2532	**0.69**
									9506	2247	**0.46**
								Panola	9502	2947	**0.54**
**Hotspot: Illinois / Kentucky**											
Core Scientific	Calvert City, KY		Shawnee Fossil Plant	West Paducah, KY	Coal		**Metropolis, IL**	Massac	9702	3679	**0.96**
Bitdeer	Knoxville, TN	→				→			9704	2293	**0.71**
Core Scientific 1^7^	Marble, NC								9701	4644	**0.41**
							**Paducah, KY**	McCracken	315.01	2524	**0.64**
									314.01	1680	**0.46**

1 Hotspots are empirically defined as contiguous counties containing at least one census tract with a total (i.e., primary and secondary) Bitcoin mine-attributable PM_2.5_ concentration ≥ 0.10 ⌈g/m^3^. See Fig. 3.

2 “Responsible Bitcoin mines” are those whose electricity consumption is primarily responsible for the Bitcoin mine-attributable PM_2.5_ concentrations in each hotspot and affected community.

3 Among plants that respond to the mines’ marginal electricity consumption, “responsible power plants” are those that contribute the largest quantity of mine-attributable PM_2.5_ over the affected communities.

4 Selected affected communities represent census tracts that are most affected by Bitcoin mine-attributable PM_2.5_ pollution. Communities listed in this table are the three census tracts within each hotspot and county with the highest Bitcoin mine-attributable PM_2.5_ concentrations above 0.4 μg/m^3^. This table is not exhaustive, and many counties have many more affected census tracts.

5 Astoria Energy refers to combined pollution from both Astoria I and Astoria II generating plants.

6 Rikers Island is a jail complex in New York City, whose inmate population fluctuated between 5,708 on August 1, 2022, to 6,182 on August 1, 2023. Estimated population does not include jail staff.^26^

7 Electricity for Core Scientific’s Bitcoin mine in Marble, NC, was supplied by two separate utilities. Only the portion supplied by TVA-Murphy Power contributed to PM_2.5_ pollution over this hotspot.

## Data Availability

The dataset we constructed for our analysis will be made available for collaboration with the authors of this work. Requests can be addressed to the first or corresponding author.

## Code availability

Code for all analyses presented here is available upon request, and it will be made publicly available on GitHub prior to publication.
